# The Effect of Fasting on Cardiovascular Diseases: A Systematic Review

**DOI:** 10.7759/cureus.53221

**Published:** 2024-01-30

**Authors:** Kirubel T Hailu, Korlos Salib, Sanath Savithri Nandeesha, Alousious Kasagga, Chnoor Hawrami, Erica Ricci, Pousette Hamid

**Affiliations:** 1 Internal Medicine, California Institute of Behavioral Neurosciences & Psychology, Fairfield, USA; 2 Internal Medicine, Afet Speciality Clinic, Addis Ababa, ETH; 3 Pathology, California Institute of Behavioral Neurosciences & Psychology, Fairfield, USA; 4 Pediatric Surgery, California Institute of Behavioral Neurosciences & Psychology, Fairfield, USA; 5 Anesthesiology, California Institute of Behavioral Neurosciences & Psychology, Fairfield, USA; 6 Neurology, California Institute of Behavioral Neurosciences & Psychology, Fairfield, USA

**Keywords:** intermittent fasting, calory deficit, prevention, cardiovascular diseases, fasting

## Abstract

Among the leading causes of morbidity, disability, and death worldwide are cardiovascular diseases (CVDs). Their risk factors usually include a variety of factors associated with cardiometabolic disorders. Many public health organizations prioritize the prevention of CVDs and encourage people to maintain a healthy lifestyle. It has been shown that fasting and a healthy diet can promote weight loss and improve cardiometabolic health in various animal species. We want to know the impact of fasting on CVDs. The topic is examined in this systematic review. We looked through a wide range of online sources, including PubMed, Cochrane Library, and Google Scholar, to find randomized controlled trials (RCTs) that looked into the connection between CVDs and fasting. We included human research that has been published in English in peer-reviewed publications in the last five years, and then we screened by the title, abstract, and full-text accessibility. We picked the final 10 articles for quality assessment using Cochrane Collaboration's tool for risk-of-bias assessment of RCTs. The findings suggest that fasting is beneficial in lowering the cardiovascular risk of a population. This result holds for all types of fasting used as an intervention in the clinical trials we reviewed. The result is pronounced when fasting regimens are combined with a regular exercise routine. More comprehensive data will come from larger-scale clinical trials and case-control studies, and a thorough examination of all the potential health impacts of fasting is warranted.

## Introduction and background

Cardiovascular diseases (CVDs) stand as the foremost cause of morbidity, disability, and fatalities globally. Their risk factors typically encompass a range of elements linked to cardiometabolic conditions [1]. The most common risk factors and associated pathologies include metabolic syndrome [2,3], obesity [4,5], hypertriglyceridemia [5,6], type 2 diabetes mellitus [7], circadian disruption [8], and dyslipidemia [5,6]. Preventing CVDs is a primary focus for numerous public health organizations, advocating for individuals to uphold a healthy lifestyle. The American Heart Association (AHA) and the American College of Cardiology (ACC) have released a comprehensive report emphasizing CVD prevention strategies [9].

Fasting is a broad term encompassing intermittent fasting (IF) [6], dietary protein restriction [2], energy restriction [3], continuous calorie restriction (CCR) [5], time-restricted eating (TRE) [8], and calorie restriction (CR) [10]. Fasting while ensuring adequate nutrition has demonstrated its effectiveness in aiding weight loss and enhancing cardiometabolic health across diverse animal species. However, in human studies, the impact of fasting on CVDs has been a topic of debate and remains inadequately comprehended. In this document, we will systematically review clinical trials to study the effect of different types of fasting on CVDs; this review seeks to provide a comprehensive understanding of the potential role of fasting in preventing and managing CVDs.

Our review is particularly timely, as the global burden of CVDs continues to escalate [11,12,13], and there is a growing interest in lifestyle modifications as a means to combat this trend [14,15]. The insights gained from this systematic review could have significant implications for clinical practice and public health policies, offering an alternative approach to CVD management that is grounded in lifestyle change.

## Review

Methodology

This systematic review utilized the Preferred Reporting Items for Systematic Review and Meta-analysis (PRISMA) guidelines [16]. The articles were systematically searched in PubMed, Google Scholar, and Cochrane Library trials. Only items that were published in English were taken into account. The most recent search was conducted on October 29, 2023. The following search terms were used in combination: intermittent fasting OR fasting OR water fasting OR autophagy, calory restriction OR time-restricted feeding OR 16/8 fasting OR 5:2 diet OR eat-stop-eat OR warrior diet OR hunger OR extended fasting. The search was limited to studies published between 2019 and 2023. In addition, the search inclusion in PubMed Medical Subject Headings (MeSH) is shown in Table 1:

**Table 1:** PubMed Medical Subject Heading (MeSH), Cochrane, and Google Scholar strategies

Database	Strategies	Number
PubMed	Intermittent fasting OR fasting OR water fasting OR Autophagy, calory restriction OR Time-restricted feeding OR 16/8 fasting OR 5:2 diet OR eat-stop-eat OR warrior diet OR hunger OR extended fasting OR (( "Intermittent Fasting/adverse effects"[Majr] OR "Intermittent Fasting/blood"[Majr] OR "Intermittent Fasting/metabolism"[Majr] OR "Intermittent Fasting/physiology"[Majr] )) AND ( "Intermittent Fasting/adverse effects"[Majr:NoExp] OR "Intermittent Fasting/blood"[Majr:NoExp] OR "Intermittent Fasting/metabolism"[Majr:NoExp] OR "Intermittent Fasting/physiology"[Majr:NoExp] ) AND Cardiovascular diseases OR coronary heart disease OR (("Cardiovascular Diseases/prevention and control"[Majr]) AND "Cardiovascular Diseases/prevention and control"[Mesh:NoExp]) OR "Cardiovascular Diseases/prevention and control"[Majr:NoExp])	392,366
Cochrane	Fasting and cardiovascular diseases	60
Google scholar	Fasting and cardiovascular diseases	12

Eligibility Criteria and Study Selection

We conducted a thorough evaluation of the title and abstract of each paper to ascertain its suitability. The results were screened using the following inclusion criteria: 1) full articles that are freely accessible, 2) studies published in the English language in the past five years, 3) peer-reviewed randomized controlled trials (RCTs) that explore the relationship between fasting and CVDs, and 4) studies conducted on human irrespective of age, ethnicity, or study location. The exclusion criteria were 1) editorials, posters, and animal research, and 2) gray literature was omitted from the analysis. Only studies that met the specified criteria were considered for eligibility in the final review.

*Data Extraction* 

We conducted data extraction from the chosen studies. The standardized recording tool was used to investigate the following variables: study design, number of study participants, baseline characteristics of participants, fasting type and treatment duration, mean follow-up in each group of participants, study outcomes, and funding source (pharmaceutical company or not).

*Quality Assessment Tool* 

We assessed the potential for bias by utilizing the Cochrane risk-of-bias method and only included studies judged as “low-risk” bias in each domain. Disagreement was resolved by consensus. 

Results 

**Figure 1:**
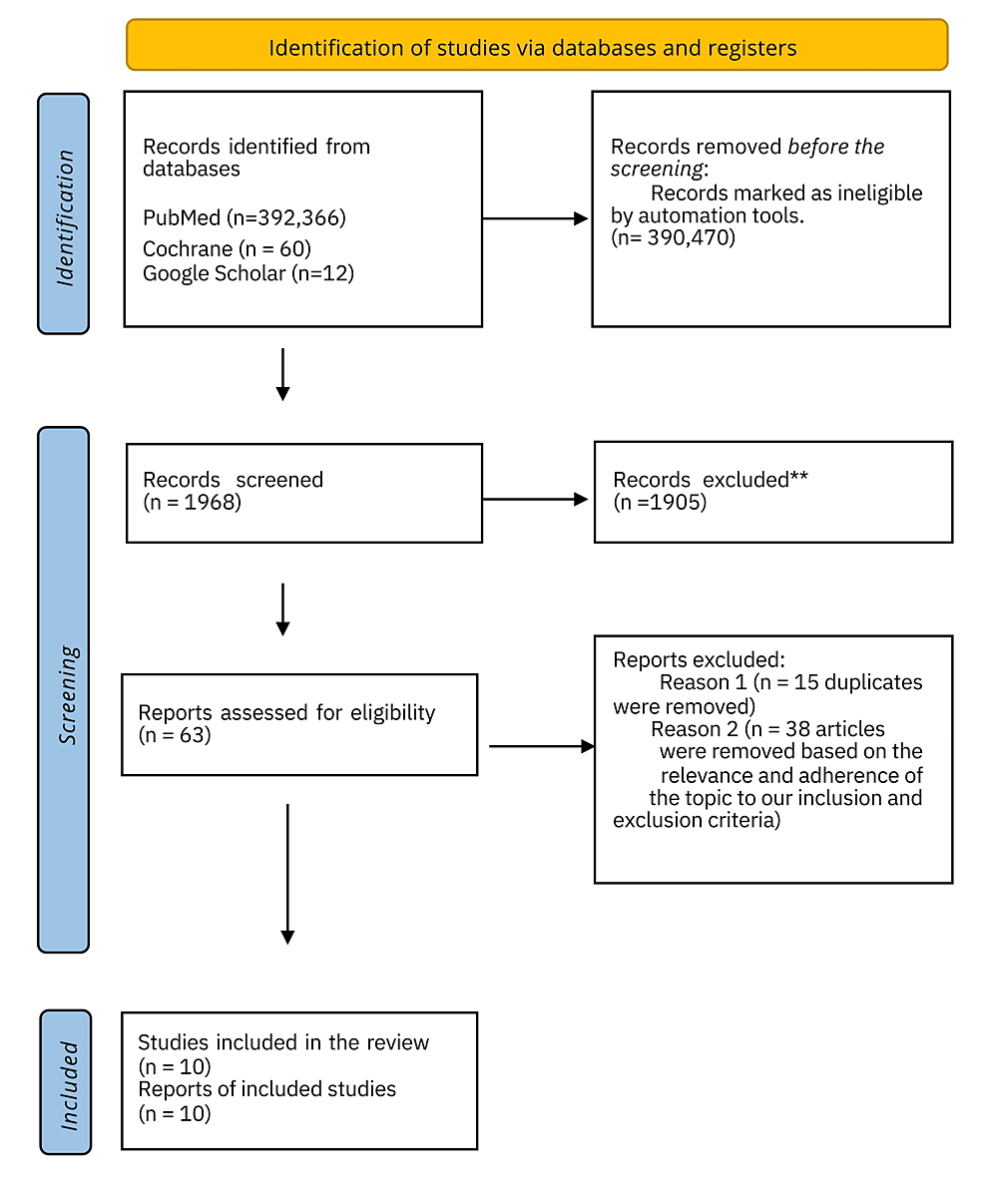
Preferred Reporting Items for Systematic Review and Meta-analysis (PRISMA) 2020 flow diagram

Upon thoroughly searching the databases, we obtained a total of 1,968 articles. Subsequently, we meticulously examined and evaluated the titles, resulting in the selection of 101 research. After doing a thorough review of the articles and eliminating 15 instances of repetition (10 articles from PubMed and five articles from other sources), we were left with a total of 10 articles (as depicted in Figure 1).

We employed Cochrane quality assessment instruments for the remaining 10 papers after comprehensively examining all articles. Table 2 displays the assessment for the 10 articles.

**Table 2:** Cochrane Collaboration's tool for risk-of-bias assessment of randomized controlled trials Risk of bias: L = low, H = high, U = unclear

	Tang et al. [1]	Ferraz-Bannitz et al. [2]	Kunduraci and Ozbek [3]	Schroder et al. [4]	Maroofi and Nasrollahzadeh [5]	Ahmed et al. [6]	Chair et al. [7]	Manoogian et al. [8]	Kraus et al. [10]	Moro et al. [11]
Random sequence generation?	L	L	H	L	L	H	L	L	L	L
Blinding?	H	L	L	L	H	L	L	H	H	H
Concurrent therapies similar?	H	H	H	H	H	H	U	H	H	H
Incomplete outcome data addressed?	H	H	H	H	H	U	U	H	H	H
Uniform and explicit outcome definitions?	H	H	H	H	H	H	H	H	H	H
Free of selective outcome reporting?	H	H	L	H	H	L	H	H	H	H
Free of other bias?	H	L	L	U	H	U	L	H	H	H
Overall risk of bias?	L	L	L	L	L	L	L	L	L	L

Table 3 displays the key characteristics and conclusions of the 10 articles. 

**Table 3:** Characteristics of studies included in this system review HDL: high-density lipoprotein, CALERIE: Comprehensive Assessment of the Long-Term Effects of Reducing Intake of Energy

Author	Journal and publication year	Title	Conclusion	Type of research
Tang et al. [1]	*Nutrients*, 2021	“Effects of Caloric Restriction and Rope-Skipping Exercise on Cardiometabolic Health”	In young adults, calorie restriction and rope-skipping exercise can both help with weight loss and body composition improvement.	A pilot randomized controlled trial in young adults
Ferraz-Bannitz et al. [2]	*Nutrients*, 2022	“Dietary Protein Restriction Improves Metabolic Dysfunction in Patients with Metabolic Syndrome”	The main factor underpinning the advantages of diet restriction is protein.	Randomized controlled trial
Kunduraci and Ozbek [3]	*Nutrients*, 2020	“Does the Energy Restriction Intermittent Fasting Diet Alleviate Metabolic Syndrome Biomarkers?”	No significant differences were observed in metabolic syndrome biomarkers between the Intermittent and Continuous energy restriction groups.	Randomized controlled trial
Schroder et al. [4]	*Journal of Translational Medicine*, 2021	“Effects of time-restricted feeding in weight loss, metabolic syndrome and cardiovascular risk in obese women”	A time-restricted feeding regimen lowers body weight without altering metabolic syndrome-related biomarkers.	Non-randomized controlled trial
Maroofi and Nasrollahzadeh [5]	*Lipids in Health and Disease*, 2020	“Effect of intermittent versus continuous calorie restriction on body weight and cardiometabolic risk markers in subjects with overweight or obesity and mild-to-moderate hypertriglyceridemia”	A three-day-per-week intermittent caloric restriction diet is similar to a continuous caloric restriction diet for lowering triglyceride levels in patients with hypertriglyceridemia. Additionally, short-term benefits of intermittent caloric restriction over continuous dieting include improved insulin resistance.	Randomized trial
Ahmed et al. [6]	*Frontiers in Nutrition*, 2020	“Impact of Intermittent Fasting on Lipid Profile”	By improving the lipid profile and raising the high-density lipoprotein (HDL), intermittent fasting may safeguard cardiovascular health.	Quasi-randomized clinical trial
Chair et al. [7]	*Journal of Nursing Research*, 2022	“Intermittent Fasting in Weight Loss and Cardiometabolic Risk Reduction”	The results suggest that incorporating intermittent fasting regimens into regular eating patterns may be beneficial in lowering the population's risk of diabetes and cardiovascular disease.	Randomized controlled trial
Manoogian et al. [8]	*Cell Metabolism*, 2022	“Feasibility of time-restricted eating and impacts on cardiometabolic health in 24-h shift workers”	There were no notable variations found in metabolic syndrome indicators between the groups that underwent intermittent and continuous calorie restriction.	Randomized control trial
Kraus et al. [10]	*Lancet Diabetes Endocrinol*, 2019	“2 years of calorie restriction and cardiometabolic risk comprehensive Assessment of Long-term Effects of Reducing intake of Energy (CALERIE): exploratory outcomes of a multicentre.”	The results hold promise for significant long-term population health benefits and point to a possible significant benefit of moderate calorie restriction for cardiovascular health in young and middle-aged healthy adults.	Phase 2, randomized controlled trial
Moro [11]	*Medicine and Science in Sports and Exercise*, 2021	“Twelve Months of Time-restricted Eating and Resistance Training Improves Inflammatory Markers and Cardiometabolic Risk Factors”	Our findings imply that a resistance exercise program in conjunction with a long-term time restriction on eating is a realistic, safe, and efficient way to lower inflammatory markers and risk factors associated with metabolic and cardiovascular disorders.	Single-blind randomized study

The clinical trials in our study have used different types of fasting as an intervention in conducting the trials: The first one is IF [5,6,7]; the second is a dietary restriction from calories, protein, or energy [1,2,3,10], and the last one is time-restricted feeding [4,8,11].

Body weight loss, waist circumference reduction, and improved lipid profiles are all possible outcomes of IF [6]. The method of fasting used here was fasting for approximately 12 hours throughout the day, from 6 A.M. to 6 P.M., three days a week for six weeks [6]. Compared to other types of IF, this method seems safe, successful, and feasible to incorporate into regular life without causing additional financial or physical strain. With IF, people do not have to worry about making extra effort to cook meals that are low in calories. A reasonable dinnertime and an early breakfast can help you keep the 12-hour fast, and this strategy works both on weekdays and weekends. It could be challenging for those with night-shift jobs, a busy social schedule, or a regular dining-out schedule. This was also seen in the current trial when five participants left because they could not keep up a three-day weekly fast due to their demanding schedules [6]. One of the studies investigated the two IF strategies, alternate-day fasting, and 16/8 time-restricted fasting. The results showed that during the trial, both intervention groups considerably outperformed the control group regarding lowering body weight, BMI, and waist circumference. Over time, the 16/8 time-restricted fasting group did not achieve as large of reductions in body weight and BMI as the alternate-day fasting group did [7].

The effect of IF versus continuous fasting on blood triglyceride levels was assessed. The trial's findings showed that intermittent CR three days a week is equivalent to a continuous energy restriction diet for lowering plasma triglyceride levels. Moreover, it seemed more successful than continuous dieting at improving a marker of insulin resistance in this group of hypertriglyceridemia patients [5]. The two cohorts had no discernible variation in plasma lipids and lipoproteins. Both diets showed beneficial modifications in lipid levels. Our investigation indicated that intermittent CR reduced plasma triglyceride levels. In terms of plasma high-density lipoprotein cholesterol (HDL-C) concentration, at eight weeks, there was no statistically significant difference between the groups; however, the CCR group's concentration dropped while the intermittent CR group's concentration remained unchanged. Since there was no drop in the ratio of HDL compared to the total cholesterol (TC/HDL-C), it is doubtful that this decrease is harmful. Insulin and the homeostasis model assessment of insulin resistance (HOMA-IR), a measure of insulin resistance, showed positive modulations under the intermittent CR diet [5].

The clinical outcomes of a 12-week intermittent fasting treatment in individuals with metabolic syndrome are reported for the first time. Intermittent energy restriction was an effective intervention that resulted in a mean weight loss of 8%, a decreased waist/hip ratio, and no negative side effects. All participants saw improvement in every metabolic indicator except HDL. Regarding alterations in the patients' glycemic readings, lipid profile, and blood pressure, the intermittent energy-restriction diet was not substantially better than the continuous calorie-restricted diet [3].

For the first time, a two-year multicenter randomized clinical trial examined the course of cardiometabolic adaptations to two years of moderate CR in healthy, non-obese young and middle-aged people with clinically normal risk factors at baseline. The trial's findings showed that even in healthy young and middle-aged individuals with normal baseline values, CR combined with adequate nutrition improves multiple cardiometabolic risk factors. Moreover, they discovered that CR in this population improved previously normal risk factors, suggesting improvement in long-term cardiovascular risk. Furthermore, these findings show that maintaining CR for two years positively impacts cardiometabolic health in addition to those brought about by the corresponding reduction in body weight. No pharmaceutical medication has such a dramatic influence on such a wide spectrum of cardiometabolic risk variables. These ought to give medical professionals a new weapon in their arsenal against the devastations of the American way of life in the 21st century [10].

They demonstrated the numerous clinical benefits that patients with metabolic syndrome who undergo short-term (i.e., 27-day) protein restriction or CR interventions appreciate, such as decreased adiposity, blood pressure normalization, improved insulin sensitivity, decreased glucose and lipid levels, and decreased systemic inflammation. The effects of protein restriction or CR last for at least a month following hospital discharge. We discovered that improving a number of metabolic metrics does not need CR. Rather than requiring a decrease in calorie intake, protein restriction is adequate to provide nearly the same clinical results as CR [2].

The protein and CR diets achieved weight loss, mainly due to decreased fat mass and preservation of free fat mass, despite the disparities in calorie intake between the two regimes. The findings demonstrated that individuals with metabolic syndrome who underwent 27 days of protein or CR had a normalization of their blood pressure and a decrease in the levels of circulating lipids, indicating that the diets under evaluation may be able to reduce the risk of CVDs. Our findings suggest that dietary protein restriction may be a workable blood pressure control method, even though it is well-established that CR lowers risk factors linked to the development of CVDs, such as high blood pressure and heart rate [2].

Protein restriction resulted in improvements in insulin sensitivity and beta-cell function (as measured by HOMA-Beta) and decreases in blood glucose and hemoglobin A1c (HbA1c) levels. These findings imply that dietary proteins might be crucial for maintaining glucose homeostasis in metabolic syndrome patients. Since eating behavior is connected to social relationships and produces enjoyment, long-term compliance with CR interventions is considered poor. This obstacle might be more difficult for obese people to overcome. This research proposes an alternate eating regimen that does not involve CR to assist people with metabolic syndrome in losing weight and managing their blood pressure, cholesterol, and blood sugar levels [2].

A separate investigation examined the short-term impacts of CR, rope-skipping (RS) exercise, and combined therapies on young adults' cardiometabolic health. While there was no discernible difference between the three groups, we discovered that the combined CR and CR-RS intervention could significantly lower weight, BMI, body fat index, and body fat mass in young people. In addition, the CR-RS combined intervention could dramatically lower low-density lipoprotein concentration (LDL-C) and interleukin eight (IL-8) levels. Crucially, the results of the subgroup analysis for the participants who were already overweight or obese at baseline demonstrated that the CR-RS combination intervention was more effective than either CR or RS in raising the levels of insulin, HOMA-IR, IL-8, systolic blood pressure (SBP), and diastolic blood pressure (DBP). More importantly, the current study demonstrated that combining CR and exercise could have more protective effects on young adults' CV health. The study also confirmed the preventative benefits of CR and exercise on the cardiovascular health of young adults [1].

The first RCT of TRE focused on those involved in 24-hour shift work, including firefighters who have a higher susceptibility to cardiometabolic illnesses. The study found that a 10-hour TRE intervention was feasible in firefighters on 24-hour shifts, with participants showing adherence to the eating window and occasional eating outside the designated window to accommodate extended evening or night calls. The study observed remarkable changes in activity, sleep, and quality of life, with a significant decrease in self-reported sleep disturbances in the TRE arm and improvements in subjective views of sleep and health. Subgroup analyses conducted on participants with elevated cardiometabolic disease risks indicated that TRE may have a more pronounced and rapid effect on persons who are at a higher risk for developing cardiometabolic disease. The study estimates that the outcomes of the TRE intervention will effectively transfer to real-life situations, specifically benefiting persons who work in shifts and those with atypical sleep-wake patterns [8].

In addition, one research examined the impact of a 12-month long-term TRE regimen on weight management and cardiovascular and metabolic risk factors in the context of a resistance exercise routine. After 12 months of TRE, the body mass was reduced by 3.4%, and inflammatory markers, lipid profile, and insulin resistance significantly improved compared with a normal diet (ND) without affecting muscle performance [11]. However, this improvement in blood biomarkers associated with metabolic and cardiovascular risk was not seen in a study to evaluate the short-term effects of time-restricted feeding. Even with short-term interventions, TRF was proven beneficial in promoting weight loss and modifications in body composition [4].

Despite the comprehensive data, our systematic evaluation has inherent limitations. We exclusively incorporated data published in English within the past five years. We specifically omitted articles that were not randomized trials. We exclusively incorporated clinical studies involving human subjects, excluding gray literature and non-peer-reviewed materials. Some of the research mentioned may suffer from recollection bias. We need further research on the effect of fasting on preventing CVDs. With the knowledge that we have right now, incorporating different forms of fasting in diet plans should be encouraged to decrease the alarming rise in the burden of CVDs.

## Conclusions

Fasting and CVDs have a close relationship. We have evidence suggesting that fasting is beneficial in lowering the cardiovascular risk of a population. This is achieved by improving the lipid profile, improving metabolic syndrome indicators, improving insulin resistance, and lowering body weight and inflammatory biomarkers. These results hold for the different types of fasting, i.e., IF, continuous fasting, CR, protein restriction, and time-restricted eating. The fact that no pharmaceutical medication has such a dramatic influence on such a wide spectrum of cardiometabolic risk variables makes these findings revolutionary. The result is pronounced when fasting regimens are combined with a regular exercise routine. Additional clinical trials and case-control studies, including a bigger sample size, are necessary to obtain more comprehensive data before reaching a definitive conclusion. It is imperative that we thoroughly investigate all potential health consequences of fasting.
